# Enteroaggregative *Escherichia coli* as etiological agent of endemic diarrhea in Spain: A prospective multicenter prevalence study with molecular characterization of isolates

**DOI:** 10.3389/fmicb.2023.1120285

**Published:** 2023-03-20

**Authors:** María Teresa Llorente, Raquel Escudero, Raquel Ramiro, María Antonia Remacha, Rocío Martínez-Ruiz, Fátima Galán-Sánchez, Mónica de Frutos, Matilde Elía, Isabel Onrubia, Sergio Sánchez

**Affiliations:** ^1^Reference and Research Laboratory on Food and Waterborne Bacterial Infections, National Center for Microbiology, Institute of Health Carlos III, Madrid, Spain; ^2^Reference and Research Laboratory on Special Pathogens, National Center for Microbiology, Institute of Health Carlos III, Madrid, Spain; ^3^Servicio de Microbiología Clínica, Complejo Asistencial Universitario de León, León, Spain; ^4^Servicio de Microbiología y Parasitología, Hospital Puerta de Hierro Majadahonda, Majadahonda, Spain; ^5^Servicio de Microbiología, Hospital Universitario Puerta del Mar, Cádiz, Spain; ^6^Servicio de Microbiología, Hospital Universitario del Río Hortega, Valladolid, Spain; ^7^Servicio de Microbiología Clínica, Hospital Universitario de Navarra, Pamplona, Spain; ^8^Pediatría, Centro de Salud Valle de la Oliva, Majadahonda, Spain

**Keywords:** enteroaggregative *Escherichia coli*, diarrheagenic *Escherichia coli*, non-travel related diarrhea, children, whole-genome sequencing, molecular characterization, extraintestinal infection

## Abstract

**Background:**

Enteroaggregative *Escherichia coli* (EAEC) is increasingly associated with domestically acquired diarrheal episodes in high-income countries, particularly among children. However, its specific role in endemic diarrhea in this setting remains under-recognized and information on molecular characteristics of such EAEC strains is limited. We aimed to investigate the occurrence of EAEC in patients with non-travel related diarrhea in Spain and molecularly characterize EAEC strains associated with illness acquired in this high-income setting.

**Methods:**

In a prospective multicenter study, stool samples from diarrheal patients with no history of recent travel abroad (*n* = 1,769) were collected and processed for detection of EAEC and other diarrheagenic *E. coli* (DEC) pathotypes by PCR. An additional case–control study was conducted among children ≤5 years old. Whole-genome sequences (WGS) of the resulting EAEC isolates were obtained.

**Results:**

Detection of DEC in the study population. DEC was detected in 23.2% of patients aged from 0 to 102 years, with EAEC being one of the most prevalent pathotypes (7.8%) and found in significantly more patients ≤5 years old (9.8% vs. 3.4%, *p* < 0.001). Although not statistically significant, EAEC was more frequent in cases than in controls. WGS-derived characterization of EAEC isolates. Sequence type (ST) 34, ST200, ST40, and ST10 were the predominant STs. O126:H27, O111:H21, and O92:H33 were the predominant serogenotypes. Evidence of a known variant of aggregative adherence fimbriae (AAF) was found in 89.2% of isolates, with AAF/V being the most frequent. Ten percent of isolates were additionally classified as presumptive extraintestinal pathogenic *E. coli* (ExPEC), uropathogenic *E. coli* (UPEC), or both, and belonged to clonal lineages that could be specifically associated with extraintestinal infections.

**Conclusion:**

EAEC was the only bacterial enteric pathogen detected in a significant proportion of cases of endemic diarrhea in Spain, especially in children ≤5 years old. In particular, O126:H27-ST200, O111:H21-ST40, and O92:H33-ST34 were the most important subtypes, with all of them infecting both patients and asymptomatic individuals. Apart from this role as an enteric pathogen, a subset of these domestically acquired EAEC strains revealed an additional urinary/systemic pathogenic potential.

## 1. Introduction

Diarrheal disease is a significant cause of hospitalization and economic losses due to sick leave in developed countries (Guarino et al., 2012; Ridderstedt et al., 2018). The etiological agents include a wide range of bacteria, viruses, and parasites. Among bacterial pathogens, strains of *Escherichia coli* that cause diarrhea in humans are known collectively as diarrheagenic *E. coli* (DEC) and traditionally classified into individual pathotypes, with Shiga toxin-producing *E. coli* (STEC), enteroaggregative *E. coli* (EAEC), enteropathogenic *E. coli* (EPEC), enterotoxigenic *E. coli* (ETEC), and enteroinvasive *E. coli* (EIEC) being the most important ones (Kaper et al., 2004).

Concretely, EAEC strains colonize the intestinal mucosa *via* the aggregative adherence fimbriae (AAF), which include five variants (designated I-V) (Nataro et al., 1993; Czeczulin et al., 1997; Bernier et al., 2002; Boisen et al., 2008; Jønsson et al., 2015) and are transcriptionally regulated by the AggR activator (Elias et al., 1999; Boisen et al., 2008). AggR promotes the expression of both chromosomal and plasmid-encoded EAEC virulence factors and is therefore considered as the central regulator of virulence functions in EAEC (Dudley et al., 2006; Harrington et al., 2006). Two examples of genes commonly found in EAEC that are regulated by AggR include *aatA*, encoding a component of the dispersin transport system, and *aaiC*, encoding a type VI secretion system (Dudley et al., 2006; Harrington et al., 2006; Petro et al., 2020). Strains harboring the AggR regulon or its components have been termed typical EAEC (Nataro, 2003), and many studies have strongly associated them with diarrhea (Wilson et al., 2001; Pabst et al., 2003; Cohen et al., 2005; Denno et al., 2012). These strains were the focus of our work and therefore, from now on, the term EAEC will specifically refer to “typical EAEC.” Strains showing an aggregative-adherence pattern but not carrying AggR-regulated genes are termed atypical EAEC (Nataro, 2003), and they are considered of uncertain pathogenicity (Tokuda et al., 2010; Boisen et al., 2020), despite having been isolated from food-borne outbreaks of gastrointestinal illness (Itoh et al., 1997). Additionally, EAEC strains often harbor a variable number of serine protease autotransporters of the *Enterobacteriaceae* (SPATEs) (Boisen et al., 2009).

Although often underdiagnosed, EAEC is frequently detected in both symptomatic and asymptomatic children in developing countries (Rogawski et al., 2017; Manhique-Coutinho et al., 2022) and considered one of the leading causes of travelers’ diarrhea (Estrada-Garcia and Navarro-Garcia, 2012). Additionally, there is increasing evidence that EAEC is also associated with domestically acquired diarrheal episodes in high-income countries, particularly among children (Pabst et al., 2003; Shazberg et al., 2003; Cohen et al., 2005; Nataro et al., 2006; Tobias et al., 2015). However, the specific role of EAEC in endemic diarrhea in industrialized countries remains under-recognized. In Spain, as EAEC infections are not notifiable and no surveillance has been conducted to date, the actual burden of disease is unknown, apart from several studies dealing with the etiology of travelers’ diarrhea (Vargas et al., 1998; Palmeiro et al., 2012). To better understand the importance of EAEC as etiological agent of endemic diarrhea in Spain we undertook a prospective study to investigate its occurrence in 1,769 patients with non-travel related diarrhea. Additionally, we investigated the clinical significance of EAEC infections especially among children ≤5 years old, by comparing EAEC prevalence in children with diarrhea (*n* = 256) and in healthy controls (*n* = 133). Furthermore, we performed whole-genome sequencing (WGS) on the resulting isolates (*n* = 120) to determine the molecular characteristics of EAEC strains associated with illness acquired in this setting.

## 2. Materials and methods

### 2.1. Study design and sample collection

A prospective multicenter study was performed from June 2015 to December 2016 in collaboration with five public tertiary hospitals located in the provinces of Madrid (central Spain), Navarra (northern Spain), Cádiz (southern Spain), Valladolid (central-western Spain), and León (north-western Spain). The collaborating laboratories were asked to submit unduplicated fresh stools from patients of any age with diarrhea and no history of recent travel abroad testing negative to other bacterial enteric pathogens after microbiologic examination. Our case definition included patients with diarrhea, either acute (≥3 liquid or semi-liquid stools in 24 h, or at least one with presence of mucus, blood, or pus for up to 2 weeks) or chronic (>4 weeks duration with decreased consistency and increased stool frequency). Cases were recruited from the emergency department, inpatient, and outpatient clinics. The samples were collected according to availability and submitted to the National Center for Microbiology (NCM, Majadahonda, Spain) once a week, up to a minimum of 350 samples per hospital. Before being shipped to NCM, all stools were routinely tested at their respective home laboratory for *Salmonella* spp., *Shigella* spp., *Campylobacter* spp., *Yersinia enterocolitica*, and *Aeromonas* spp. (but not for DEC) using conventional microbiological methods.

We conducted an additional case–control study among children ≤5 years old living in one of the provinces included in the prospective prevalence study (Madrid). The study population consisted of all the children ≤5 years old with diarrhea for whom occurrence of DEC had been previously investigated over the prospective prevalence study from June 2015 to February 2016 (cases) (*n* = 256), and a group of randomly selected children ≤5 years old with no history of diarrhea or use of antibiotics for at least 14 days and no history of recent travel abroad (controls) (*n* = 133). These unpaired control subjects were recruited from June 2016 to February 2017 during primary care pediatric consultations in a health center belonging to the same health care district than the hospital that had provided the cases.

### 2.2. Ethical statement

Since the prospective multicenter study was approved as a part of the routine diagnostic practice, neither specific approval of the respective hospital ethics committees nor informed consent from patients was needed. As for control subjects, ethical approval and permission for the study was obtained from the health care district management (Comisión Central de Investigación, Gerencia Asistencial de Atención Primaria, Servicio Madrileño de Salud; date: May 11, 2016/Reference: 03/2016) and written informed consent was obtained from parents/legal guardians.

### 2.3. Microbiological analysis

Upon receipt, a stool impregnated cotton swab was inoculated in 5 mL of buffered peptone water (BPW, Oxoid, Basingstoke, United Kingdom) and overnight incubated at 37°C. After this non-selective enrichment step, the BPW culture was subcultured on both MacConkey agar (MAC, Becton Dickinson, Franklin Lakes, NJ, United States) and tryptic soy agar (TSA, Becton Dickinson) and overnight incubated at 37°C. A loopful of bacterial growth taken from the first streaking area of the TSA plate was suspended in 0.5 mL of sterile distilled water, boiled for 5 min to release the DNA, and centrifuged at 10,000 rpm for 5 min.

The supernatant was used directly as a template in eight in-house conventional PCR assays for the specific amplification of genes defining each DEC pathotype (Table 1), using DreamTaq DNA Polymerase (Thermo Fisher Scientific, Waltham, MA, USA), according to the manufacturer’s instructions. An additional *gapA*-specific PCR was also run concurrently with the diagnostic PCR assays to ensure that all samples had sufficient bacterial DNA present and no PCR inhibition occurred (Table 1). Thermal cycler conditions consisted of 25 cycles of denaturation at 94°C for 30 s, annealing at 56°C for 40 s, and extension at 72°C for 1 min. Our diagnostic criterion for EAEC infection was the presence of *aatA* gene, considering its historical specificity (Pabst et al., 2003; Beczkiewicz et al., 2019). According to this criterion, the study focused only on typical EAEC. The diagnostic criteria for other DEC infections were as follows: for STEC, the presence of *stx1*, and/or *stx2*, and/or *stx2f*, and possible additional gene *eae*, with STEC primers targeting the specific subtypes *stx1a*, *stx1c*, *stx1d*, *stx2a*, *stx2b*, *stx2c*, *stx2d*, *stx2e*, *stx2f*, and *stx2g*; for EPEC, the presence of *eae* and possible additional gene *bfpA*, with the absence of *bfpA* indicating atypical EPEC (aEPEC); for ETEC, the presence of *eltA* and/or *estA*; for EIEC, the presence of *ipaH*.

**Table 1:** Primer pairs and target genes used for detection and isolation of diarrheagenic *Escherichia coli* pathotypes in patients with endemic diarrhea and asymptomatic controls.

Pathotypes^a^	Target gene	Primer^b^	PCR assay^c^	Oligonucleotide sequence (5′-3′)	Product size (bp)	References
STEC	*stx1*	VT1a	1	GAAGAGTCCGTGGGATTACG	130	Pollard et al. (1990)
		VT1b		AGCGATGCAGCTATTAATAA		
	*stx2*	SLTII-1	1	CTTCGGTATCCTATTCCCGG	478	Olsen et al. (1995)
		SLTII-2		GGATGCATCTCTGGTCATTG		
	*stx2f*	vtx2f-F1	2	TGGGCGTCATTCACTGGTTG	424	Scheutz et al. (2012)
		vtx2f-R1		TAATGGCCGCCCTGTCTCC		
STEC, EPEC	*eae*	SK1	3	CCCGAATTCGGCACAAGCATAAGC	881	Oswald et al. (2000)
		SK2		CCCGGATCCGTCTCGCCAGTATTCG		
tEPEC	*bfpA*	EP1	4	AATGGTGCTTGCGCTTGCTGC	326	Gunzburg et al. (1995)
		EP2		GCCGCTTTATCCAACCTGGTA		
EAEC	*aatA*	pCDV432/start	5	CTGGCGAAAGACTGTATCAT	630	Schmidt et al. (1995)
		pCDVD432/stop		CAATGTATAGAAATCCGCTGTT		
ETEC	*eltA*	LT-A-1	6	GGCGACAGATTATACCGTGC	696	Schultsz et al. (1994)
		LT-A-2		CCGAATTCTGTTATATATGTC		
	*estA*	stIaF	7	TTTCCCCTCTTTTAGTCAGTC	159/138 ^d^	Modified from Guion et al. (2008)
		stIbF		TGCTAAACCAGTAGAGTCTTC		Modified from Guion et al. (2008)
		stR		GCAGGATTACAACACAATTCACAGCAG		Guion et al. (2008)
EIEC	*ipaH*	EI1	8	GCTGGAAAAACTCAGTGCCT	424	Tornieporth et al. (1995)
		EI2		CCAGTCCGTAAATTCATTCT		
Control^e^	*gapA*	gapA-F	9	ATCAACGGTTTTGGCCGTATC	924	This study
		gapA-R		GTTGTCGTACCAGGAYACCAG		

STEC, Shiga toxin-producing *E. coli*; EPEC, enteropathogenic *E. coli*; tEPEC, typical EPEC; EAEC, enteroaggregative *E. coli*; ETEC, enterotoxigenic *E. coli*; EIEC, enteroinvasive *E. coli*.

a The criteria for diagnosis of a diarrheagenic *E. coli* (DEC) infection were as follows: for EAEC, the presence of *aatA*; for STEC, the presence of *stx1*, and/or *stx2*, and/or *stx2f*, and possible additional gene *eae*; for EPEC, the presence of *eae* and possible additional gene *bfpA*, with the presence of *bfpA* indicating tEPEC and the absence indicating atypical EPEC; for ETEC, the presence of *eltA* and/or *estA*; for EIEC, the presence of *ipaH*.

b All PCR reactions contained 200 nM of each primer.

c Thermal cycler conditions consisted of 25 cycles of denaturation at 94°C for 30 s, annealing at 56°C for 40 s, and extension at 72°C for 1 min.

d PCR amplification with primers stIaF/stR and stIbF/stR targeted ST-Ia (or STh) and ST-Ib (or STp), respectively, which are the variants of the heat stable enterotoxin involved in human disease, and yielded products of 159 bp and 138 bp, respectively.

e Additional *gapA*-specific PCR to ensure that all samples had sufficient bacterial DNA present and no PCR inhibition occurred.

When culture tested EAEC-positive, up to 20 individual *E. coli*-like colonies obtained from MAC plates were tested by PCR to obtain the isolate, which was further confirmed biochemically as *E. coli* by the API 20E system (bioMérieux, Marcy l’Etoile, France).

### 2.4. Whole-genome sequencing

Genomic DNA was purified from the EAEC isolates using the QIAamp DNA Mini Kit (QIAGEN, Hilden, Germany) according to the manufacturer’s instructions. A DNA library was generated using the Nextera XT DNA Sample Preparation Kit (Illumina, San Diego, CA, United States) according to the manufacturer’s instructions and run on a NextSeq 500 (Illumina) for generating paired-end 150 bp reads, aiming at a coverage of at least 200-fold. The reads were trimmed (FastP, 0.23.2) and filtered according to quality criteria (FastQC, 0.11.9), and the quality-filtered reads were *de novo* assembled by using Unicycler (v0.4.8) (Wick et al., 2017).

### 2.5. Data analysis and molecular characterization

The O and H serogenotypes (*in silico* serotypes), virulence genes, sequence types (STs), and acquired antibiotic resistance genes, were identified by uploading the reads to SerotypeFinder 2.0, VirulenceFinder 2.0, MLST 2.0, and ResFinder 4.1, respectively, available on the Center for Genomic Epidemiology (CGE) website.^1^ The threshold of sequence identity was set to 85% and the percentage of minimum overlapping gene length to 60%. MLST tool used the seven loci (*adk*, *gyrB*, *fumC*, *icd*, *mdh*, *purA*, and *recA*) scheme. When SerotypeFinder did not predict O antigen it was considered not typeable (ONT). The *E. coli* phylogroups were determined using the ClermonTyping tool available on the Iame-research Center website.^2^ The presence of the colonization factor CS22 structural gene (*cseA*, accession no. AF145205.1) was determined by searching the assembled contigs with BLASTn. The presumptive extraintestinal pathogenic *E. coli* (ExPEC) status was assigned to those isolates positive for ≥2 of the following virulence genes: *papA* and/or *papC*, *sfa-focDE*, *afa-draBC*, *iutA*, and *kpsMII* (Johnson et al., 2003). For this purpose, isolates were considered positive for *afa-draBC* if a combination of *afaB* or *nfaE* and also *afaC* was identified by WGS and positive for *sfa-focDE* if a combination of *focC* or *sfaE* and also *focI* or *sfaD* was identified (Malberg Tetzschner et al., 2020). Likewise, the uropathogenic *E. coli* (UPEC) status was assigned to those isolates positive for ≥2 of the following genes: *chuA*, *fyuA*, *vat*, and *yfcV* (Spurbeck et al., 2012; Malberg Tetzschner et al., 2020).

### 2.6. Phylogenomic analysis

The 120 EAEC genomes analyzed in this study were compared with 195 previously sequenced EAEC genomes originating from the United Kingdom, Egypt, Kenya, or Peru (Do Nascimento et al., 2017; Ellis et al., 2020; Petro et al., 2020), as well as the EAEC reference genomes 17–2, 042, and 55,989, and six ExPEC reference genomes (Supplementary Table 1) using Snippy 4.6.0 as previously described^3^. Snippy identified 690,639 conserved SNPs, compared against the reference genome of the *E. coli* strain IAI39 (accession no. NC_011750.1), that were used to infer a maximum likelihood phylogeny using IQ-Tree 2.1.4 (Minh et al., 2020) with a TVM model and 1,000 bootstrap iterations. The phylogeny was midpoint rooted and annotated with iTOL 6.6 (Letunic and Bork, 2019).

Additionally, a more specific SNP analysis was performed for each of the most important serogenotype-ST combinations identified in the present study, including isolates from both the present and previous studies (Do Nascimento et al., 2017; Ellis et al., 2020; Petro et al., 2020). The analysis was carried out by uploading the reads to CSI Phylogeny 1.4, available on the CGE website, with the following settings: a minimum depth of 10 at SNP positions, a minimum relative depth of 10% at SNP positions, a minimum distance of 10 bp between SNPs (prune), a minimum SNP quality of 30, a minimum read quality of 25, and a minimum Z-score of 1.96. According to KmerFinder 3.2 results, the published genomes of *E. coli* strains A41 (accession no. NZ_CP028735.1), ESBL 15 (accession no. NZ_CP041678.1), BR1220 (accession no. NZ_CP093068.1), and H3 (accession no. NZ_CP028732.1) were used as a reference for EAEC strains belonging to O126:H27-ST200, O111:H21-ST40, O92:H33-ST34, and O3:H2-ST10, respectively. The percentage of the reference genome covered by all isolates of the same serogenotype-ST combination ranged between 82.3 and 90.3%. From the aligned sequences of concatenated SNPs, we calculated maximum likelihood phylogenetic trees with RAxML 8.2.12 (Stamatakis, 2014) with a GTR model and 1,000 bootstrap iterations. The respective consensus trees were midpoint rooted and annotated with iTOL.

### 2.7. Sequence availability

FASTQ sequences were deposited in the National Center for Biotechnology Information Short Read Archive under the BioProject PRJNA863489. Accession numbers for each sequence are listed in Supplementary Table 2.

### 2.8. Statistical analysis

The sample size for determining the DEC prevalence in the prospective multicenter study was calculated using the website tool www.openepi.com, with a confidence level of 95%, a precision value of 3%, and an anticipated frequency of 9% (Nataro et al., 2006). The case–control study was conducted with as many healthy controls fulfilling the inclusion criteria as possible. Proportions were compared by a two-tailed chi-square test or Fisher’s exact test and odds ratios with 95% confidence intervals were determined. A *p* value <0.05 was considered statistically significant.

## 3. Results

### 3.1. Detection of diarrheagenic *Escherichia coli* in the study population

The prospective study included 1,769 patients (mean age 14.5 years, range 0–102 years, standard deviation (SD) 24.4 years, male 54%), with 68.6% of participants being ≤5 years old (*n* = 1,213). The case–control study included 256 children with diarrhea (mean age 18.1 months, SD 16.2 months, male 55.1%) and 133 asymptomatic children (mean age 16.4 months, SD 14 months, male 59.4%).

At least one DEC pathotype was detected in 23.2% (410/1,769) of patients and more than one (up to three) was detected in 2.3% (40/1,769) (Table 2). EAEC was one of the pathotypes most commonly found (7.8%, 138/1,769), second only to aEPEC (13.3%, 235/1,769) and followed by STEC (3.6%, 63/1,769), with ETEC and EIEC being anecdotal (0.6%, 10/1,769, and 0.3%, 5/1,769, respectively). The most common DEC co-infection was the combination of EAEC and aEPEC, detected in 62.5% (25/40) of co-infection episodes. There were no statistically significant differences between male and female patients neither in relation to general DEC prevalence nor in relation to EAEC (data not shown). However, DEC infection was found in significantly more patients ≤5 years old (27.7%, 336/1,213, vs. 13.3%, 74/556, *p* < 0.001), with EAEC and aEPEC infections being particularly associated with this age group (9.8%, 119/1,213, vs. 3.4%, 19/556, *p* < 0.001 for EAEC; 16.2%, 197/1,213, vs. 6.8%, 38/556, *p* < 0.001 for aEPEC; 2.1%, 25/1,213, vs. 0%, 0/556, *p* < 0.001 for EAEC+aEPEC co-infection) (Table 2).

**Table 2:** Detection of diarrheagenic *Escherichia coli* pathotypes among patients with endemic diarrhea and differences in prevalence by age.

	No. (%) of DEC-positive stool samples			Odds ratio (95% CI)	*p* value^d^
		Age group, years			
	All (*n* = 1,769)	≤5^b^ (*n* = 1,213)	>5^c^ (*n* = 556)		
Patients with any DEC	410 (23.2)	336 (27.7)	74 (13.3)	2.5 (1.9–3.3)	**<0.001**
EPEC	235 (13.3)	197 (16.2)	38 (6.8)	2.6 (1.8–3.8)	**<0.001**
tEPEC	0	0	0	NA	NA
aEPEC	235 (13.3)	197 (16.2)	38 (6.8)	2.6 (1.8–3.8)	**<0.001**
ETEC	10 (0.6)	4 (0.3)	6 (1.1)	0.3 (0.1–1.1)	NS
ST	3 (0.2)	1 (0.1)	2 (0.4)	0.2 (0.0–2.5)	NS
LT	5 (0.3)	2 (0.2)	3 (0.5)	0.3 (0.1–1.8)	NS
ST + LT	2 (0.1)	1 (0.1)	1 (0.2)	0.5 (0.0–7.3)	NS
STEC	63 (3.6)	50 (4.1)	13 (2.3)	1.8 (1.0–3.3)	NS
EIEC	5^a^ (0.3)	3 (0.2)	2 (0.4)	0.7 (0.1–4.1)	NS
EAEC	138 (7.8)	119 (9.8)	19 (3.4)	3.1 (1.9–5.0)	**<0.001**
DEC co-infections	40 (2.3)	36 (3.0)	4 (0.7)	4.2 (1.5–11.9)	**0.003**
EAEC+aEPEC	25 (1.4)	25 (2.1)	0	ND	**<0.001**
aEPEC+STEC	5 (0.3)	4 (0.3)	1 (0.2)	1.8 (0.2–16.5)	NS
EAEC+STEC	3 (0.2)	3 (0.2)	0	ND	NS
aEPEC+ETEC	2 (0.1)	0	2 (0.4)	ND	NS
EAEC+ETEC	2 (0.1)	1 (0.1)	1 (0.2)	0.5 (0.0–7.3)	NS
STEC+ETEC	1 (0.1)	1 (0.1)	0	ND	NS
EIEC+aEPEC	1 (0.1)	1 (0.1)	0	ND	NS
EAEC+ETEC+EIEC	1 (0.1)	1 (0.1)	0	ND	NS

DEC, diarrheagenic *E. coli*; EPEC, enteropathogenic *E. coli*; tEPEC, typical EPEC; aEPEC, atypical EPEC; ETEC, enterotoxigenic *E. coli*; ST, heat-stable enterotoxin-positive sample; LT, heat-labile enterotoxin-positive sample; STEC, Shiga toxin-producing *E. coli*; EIEC, enteroinvasive *E. coli*; EAEC, enteroaggregative *E. coli*; CI, confidence interval; NA, not applicable; ND, not defined; NS, not statistically significant.

Bold indicates the statistically significant *p* values.

a *ipaH*-positive isolates were not obtained from three of the samples and therefore they could not be definitely identified as EIEC or *Shigella*.

b Range 0–5 years, mean age 1.4 years, standard deviation 1.4 years.

c Range 6–102 years, mean age 43.0 years, standard deviation 26.4 years.

d Fisher’s exact test was applied when one of the observations was less than 5. A *p* value <0.05 was considered statistically significant.

In the case–control study, conducted only with children ≤5 years old, EAEC was more frequently detected in cases than in controls (12.5%, 32/256, vs. 10.5%, 14/133), although this difference was not statistically significant (Table 3). Only aEPEC and DEC co-infections in general were found in significantly more cases than controls (16.8%, 43/256, vs. 8.3%, 11/133, *p* = 0.021 for aEPEC; 5.5%, 14/256, vs. 0%, 0/133, *p* = 0.005 for DEC co-infections).

**Table 3:** Detection of diarrheagenic *Escherichia coli* pathotypes among patients with endemic diarrhea and asymptomatic controls ≤5 years old and association of specific pathotypes with diarrhea.

	No. (%) of DEC-positive stool samples		Odds ratio (95% CI)	*p* value^b^
	Children with diarrhea (*n* = 256)	Children without diarrhea (*n* = 133)		
Children with any DEC	73 (28.5)	27 (20.3)	1.6 (0.9–2.6)	NS
EPEC	43 (16.8)	11 (8.3)	2.2 (1.1–4.5)	**0.021**
tEPEC	0	0	NA	NA
aEPEC	43 (16.8)	11 (8.3)	2.2 (1.1–4.5)	**0.021**
ETEC	1 (0.4)	0	ND	NS
ST	0	0	NA	NA
LT	0	0	NA	NA
ST + LT	1 (0.4)	0	ND	NS
STEC	10 (3.9)	2 (1.5)	2.7 (0.6–12.3)	NS
EIEC	2 ^a^ (0.8)	0	ND	NS
EAEC	32 (12.5)	14 (10.5)	1.2 (0.6–2.4)	NS
DEC co-infections	14 (5.5)	0	ND	**0.005**
EAEC+aEPEC	8 (3.1)	0	ND	NS
EAEC+STEC	2 (0.8)	0	ND	NS
aEPEC+STEC	2 (0.8)	0	ND	NS
EAEC+ETEC+EIEC	1 (0.4)	0	ND	NS

DEC, diarrheagenic *E. coli*; EPEC, enteropathogenic *E. coli*; tEPEC, typical EPEC; aEPEC, atypical EPEC; ETEC, enterotoxigenic *E. coli*; ST, heat-stable enterotoxin-positive sample; LT, heat-labile enterotoxin-positive sample; STEC, Shiga toxin-producing *E. coli*; EIEC, enteroinvasive *E. coli*; EAEC, enteroaggregative *E. coli*; CI, confidence interval; NA, not applicable; ND, not defined; NS, not statistically significant.

Bold indicates the statistically significant *p* values.

a The *ipaH*-positive isolate was not obtained from one of the samples and therefore it could not be definitely identified as EIEC or *Shigella*.

b Fisher’s exact test was applied when one of the observations was less than 5. A *p* value <0.05 was considered statistically significant.

### 3.2. WGS-derived characterization of EAEC isolates

**Figure 1:**
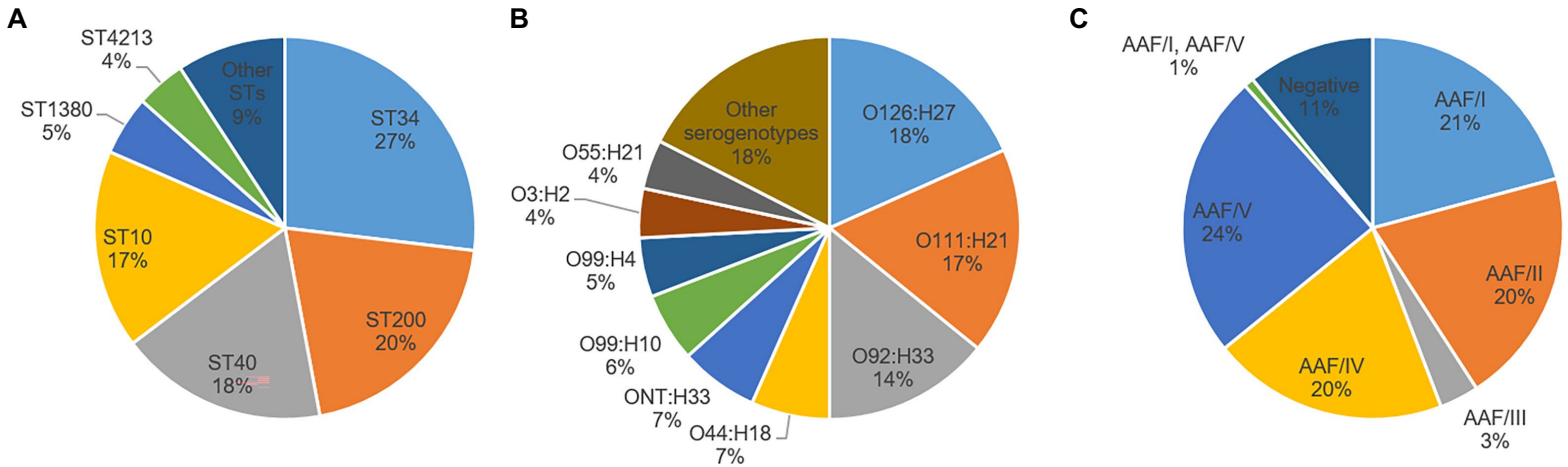
Distribution of different sequence types (ST) **(A)**, serogenotypes **(B)**, and aggregative adherence fimbriae (AAF) variants **(C)** among enteroaggregative *Escherichia coli* isolates. Other serogenotypes and STs (with less than five isolates each) are summarized in Supplementary Table 2. When O antigen was not predicted, it was considered not typeable (ONT).

EAEC was isolated from 110 (79.7%) of the 138 EAEC-positive fecal samples from patients and 10 (71.4%) of the 14 EAEC-positive samples from asymptomatic individuals. Characteristics of the 120 resulting EAEC isolates are summarized in Supplementary Table 2. Only four of the eight *E. coli* phylogroups *sensu stricto* recognized so far (A, B1, B2, C, D, E, F, and G) (Clermont et al., 2019) were represented among the 120 EAEC isolates. The predominant phylogroups were B1 (47%) and A (44%), although D (8%) and B2 (1%) were also identified (Supplementary Table 2). The presumptive ExPEC and/or UPEC status was found only in isolates belonging to phylogroups A (58%), D (33%), and B2 (9%). All isolates negative for both AAF and CS22 belonged to phylogroup A. Thirteen different STs and 26 different serogenotypes were found. The most common ST was ST34 (27%), followed by ST200 (20%), ST40 (18%), and ST10 (17%) (Figure 1A). The predominant serogenotype was O126:H27 (18%), followed by O111:H21 (17%) and O92:H33 (14%) (Figure 1B). Notably, three of the six serotypes defining the EAEC prototype strains were found in our collection (JM221 serotype O92:H33, 042 serotype O44:H18, and 17–2 serotype O3:H2) (Boisen et al., 2020).

Regarding the distribution of EAEC-associated putative virulence genes, the resulting EAEC isolates are typical EAEC in the formal definition, as they harbored at least *aatA* gene, per study protocol. Apart from *aatA*, the dispersin gene *aap* was the most commonly detected EAEC-associated gene (100%), followed by *aggR* (90.8%), the genes encoding the proteins ORF3 and ORF4 (89.2% each), *aaiC* (85%), the SPATE gene *pic* (83.3%), and the AggR-activated regulator *aar* (71.7%). Other SPATE genes detected were *sat* (41.7%), *sepA* (40.8%), and *pet* (20%) (Table 4). No statistically significant differences could be observed in the presence and frequency of specific putative virulence genes between isolates obtained from patients and those from controls (Table 4). Evidence of a known AAF variant was found in 89.2% of the isolates, with AAF/V being the most frequently observed (24%), followed by AAF/I (21%), AAF/II (20%), AAF/IV (20%), and AAFIII (3%) (Table 4, Figure 1C). Remarkably, the sequence of one isolate (4519–15) contained the genes for both AAF/I (*aggA*, *aggB*, *aggC*, and *aggD*) and AAF/V *(agg3C*, *agg3D*, and *agg5A*) variants (Supplementary Table 2). Thirteen isolates (10.8%) were negative for any genes attributed to the five known AAF variants, despite harboring *aggR* and/or other AggR-regulated genes. Notably, one of these AAF-negative isolates (2018–2015) had a gene identical (100% homologous) to *cseA* (Supplementary Table 2), indicative of the presence of the non-fimbrial ETEC colonization factor CS22, instead of AAF.

**Table 4:** Distribution of putative virulence genes among the 120 enteroaggregative *Escherichia coli* isolates obtained from patients with endemic diarrhea (cases) and asymptomatic individuals (controls).

Gene	Gene description	Pathotype	No. (%) of positive isolates			Odds ratio (95% CI)^a^
			Cases (*n* = 110)	Controls (*n* = 10)	Total (*n* = 120)	
*aafA*	AAF/II major fimbrial subunit	EAEC	22 (20.0)	2 (20.0)	24 (20.0)	1.0 (0.2–5.0)
*aaiC*	Type VI secretion protein	EAEC	92 (83.6)	10 (100)	102 (85.0)	ND
*aap*	Dispersin, antiaggregation protein	EAEC	110 (100)	10 (100)	120 (100)	ND
*aar*	AggR-activated regulator	EAEC	77 (70.0)	9 (90.0)	86 (71.7)	0.3 (0.0–2.1)
*aatA*	Dispersin transporter protein	EAEC	110 (100)	9 (90.0)	119 (99.2) ^b^	ND
*afaA*	Transcriptional regulator	ExPEC	3 (2.7)	0	3 (2.5)	ND
*afaB*	Periplasmic chaperone	ExPEC	3 (2.7)	0	3 (2.5)	ND
*afaC*	Outer membrane usher protein	ExPEC	3 (2.7)	0	3 (2.5)	ND
*afaD*	Afimbrial adhesion	ExPEC	61 (55.5)	5 (50.0)	66 (55.0)	1.2 (0.3–4.5)
*afaE*	Adhesin protein	ExPEC	2 (1.8)	0	2 (1.7)	ND
*agg3A*	AAF/III major fimbrial subunit	EAEC	3 (2.7)	1 (10.0)	4 (3.3)	0.3 (0.0–2.7)
*agg4A*	AAF/IV major fimbrial subunit	EAEC	23 (20.9)	1 (10.0)	24 (20.0)	2.4 (0.3–19.8)
*agg5A*	AAF/V major fimbrial subunit	EAEC	27 (24.5)	3 (30.0)	30 (25.0) ^c^	0.8 (0.2–3.1)
*aggA*	AAF/I major fimbrial subunit	EAEC	23 (20.9)	3 (30.0)	26 (21.7) ^c^	0.6 (0.1–2.6)
*aggR*	AraC transcriptional activator	EAEC	99 (90.0)	10 (100)	109 (90.8)	ND
*air*	Enteroaggregative immunoglobulin repeat protein	EAEC	10 (9.1)	0	10 (8.3)	ND
*astA*	EAST-1 heat-stable toxin	EAEC	56 (50.9)	7 (70.0)	63 (52.5)	0.4 (0.1–1.8)
*capU*	Hexosyltransferase homolog	EAEC	60 (54.5)	3 (30.0)	63 (52.5)	2.8 (0.7–11.4)
*cea*	Colicin E1	ExPEC	12 (10.9)	4 (40.0)	16 (13.3)	0.2 (0.0–0.7)
*celB*	Endonuclease colicin E2	EPEC	8 (7.3)	1 (10.0)	9 (7.5)	0.7 (0.1–6.3)
*chuA*	Outer membrane hemin receptor	ExPEC	11 (10.0)	0	11 (9.2)	ND
*cia*	Colicin Ia	ExPEC	9 (8.2)	0	9 (7.5)	ND
*cib*	Colicin Ib	ExPEC	1 (0.9)	0	1 (0.8)	ND
*cnf1*	Cytotoxic necrotizing factor	ExPEC	1 (0.9)	0	1 (0.8)	ND
*eilA*	*Salmonella* HilA homolog	EAEC	10 (9.1)	0	10 (8.3)	ND
*epeA*	Enterohemorrhagic *E. coli* EHEC plasmid-encoded autotransporter	STEC	1 (0.9)	0	1 (0.8)	ND
*espI*	Serine protease autotransporters of Enterobacteriaceae (SPATE)	EPEC	27 (24.5)	4 (40.0)	31 (25.8)	0.5 (0.1–1.9)
*fyuA*	Siderophore receptor	ExPEC	95 (86.4)	10 (100)	105 (87.5)	ND
*hra*	Heat-resistant agglutinin	EAEC/ExPEC	9 (8.2)	1 (10.0)	10 (8.3)	0.8 (0.1–7.1)
*iha*	Adherence protein	STEC	56 (50.9)	8 (80.0)	64 (53.3)	0.3 (0.1–1.3)
*irp2*	High molecular weight protein 2 non-ribosomal peptide synthetase	ExPEC	95 (86.4)	10 (100)	105 (87.5)	ND
*iss*	Increased serum survival	ExPEC	56 (50.9)	6 (60.0)	62 (51.7)	0.7 (0.2–2.6)
*iucC*	Aerobactin synthetase	ExPEC	58 (52.7)	7 (70.0)	65 (54.2)	0.5 (0.1–1.9)
*iutA*	Ferric aerobactin receptor	ExPEC	61 (55.5)	7 (70.0)	68 (56.7)	0.5 (0.1–2.2)
*kpsE*	Capsule polysaccharide export inner-membrane protein	ExPEC	18 (16.4)	1 (10.0)	19 (15.8)	1.8 (0.2–14.8)
*KpsMII*	Polysialic acid transport protein; Group 2 capsule	ExPEC	18 (16.4)	1 (10.0)	19 (15.8)	1.8 (0.2–14.8)
*lpfA*	Long polar fimbriae	STEC/EPEC	63 (57.3)	5 (50.0)	68 (56.7)	1.3 (0.4–4.9)
*mcbA*	Bacteriocin microcin B17	ExPEC	1 (0.9)	0	1 (0.8)	ND
*mchB*	Microcin H47 part of colicin H	STEC/EPEC	31 (28.2)	5 (50.0)	36 (30.0)	0.4 (0.1–1.5)
*mchC*	MchC protein	STEC/EPEC	31 (28.2)	5 (50.0)	36 (30.0)	0.4 (0.1–1.5)
*mchF*	ABC transporter protein MchF	STEC/EPEC	31 (28.2)	5 (50.0)	36 (30.0)	0.4 (0.1–1.5)
*mcmA*	Microcin M part of colicin H	STEC/EPEC	9 (8.2)	1 (10.0)	10 (8.3)	0.8 (0.1–7.1)
*neuC*	Polysialic acid capsule biosynthesis protein	ExPEC	2 (1.8)	0	2 (1.7)	ND
*nfaE*	Diffuse adherence fibrillar adhesin gene	ETEC/DAEC	3 (2.7)	0	3 (2.5)	ND
*ompT*	Outer membrane protease (protein protease 7)	ExPEC	31 (28.2)	3 (30.0)	34 (28.3)	0.9 (0.2–3.8)
*ORF3*	Isoprenoid biosynthesis	EAEC	97 (88.2)	10 (100)	107 (89.2)	ND
*ORF4*	Putative isopentenyl-diphosphate delta-isomerase	EAEC	97 (88.2)	10 (100)	107 (89.2)	ND
*papA*	Major pilin subunit	ExPEC	7 (6.4)	0	7 (5.8) ^d^	ND
*papC*	Outer membrane usher P fimbriae	ExPEC	7 (6.4)	0	7 (5.8)	ND
*pet*	Plasmid-encoded toxin	EAEC	22 (20.0)	2 (20.0)	24 (20.0)	1.0 (0.2–5.0)
*pic*	Serine protease autotransporters of Enterobacteriaceae (SPATE)	EAEC/EIEC	90 (81.8)	10 (100)	100 (83.3)	ND
*sat*	Secreted autotransporter toxin	EAEC/ExPEC	43 (39.1)	7 (70.0)	50 (41.7)	0.3 (0.1–1.1)
*senB*	Plasmid-encoded enterotoxin	STEC	2 (1.8)	1 (10.0)	3 (2.5)	0.2 (0.0–2.0)
*sepA*	*Shigella* extracellular protein A	EAEC/EIEC	45 (40.9)	4 (40.0)	49 (40.8)	1.0 (0.3–3.9)
*sitA*	Iron transport protein	ExPEC	20 (18.2)	0	20 (16.7)	ND
*terC*	Tellurium ion resistance protein	ExPEC	110 (100)	10 (100)	120 (100)	ND
*traT*	Outer membrane protein complement resistance	ExPEC	74 (67.3)	7 (70.0)	81 (67.5)	0.9 (0.2–3.6)
*yfcV*	Fimbrial protein	ExPEC	1 (0.9)	0	1 (0.8)	ND

EAEC, enteroaggregative *E. coli*; ExPEC, extraintestinal pathogenic *E. coli*; EPEC, enteropathogenic *E. coli*; STEC, Shiga toxin-producing *E. coli*; ETEC, enterotoxigenic *E. coli*; DAEC, diffusely adherent *E. coli*; EIEC, enteroinvasive *E. coli*; CI, confidence interval; ND, not defined.

a Fisher’s exact test was applied when one of the observations was less than 5. A *p* value <0.05 was considered statistically significant. None of the differences observed between isolates from cases and controls was statistically significant.

b Per study protocol, all EAEC isolates in this study possessed *aatA*, as was demonstrated by the diagnostic PCR assay, in spite of the lack of *aatA* detection by VirulenceFinder in one isolate.

c Both AAF/I and AAF/V genes were detected by VirulenceFinder in one isolate.

d Including mayor pilin subunits of types F16 (5 isolates), F7-2, and F13 (one isolate each).

Apart from EAEC-associated putative virulence genes, a substantial number of genes typically associated with other *E. coli* pathotypes were detected (Table 4). In particular, 27 of the 44 genes associated with ExPEC included in the CGE *E. coli* virulence gene database were detected among EAEC isolates. The most frequent ExPEC-associated genes were *terC* (100%), *fyuA* (87.5%), *irp2* (87.5%), *traT* (67.5%), *iutA* (56.7%), *afaD* (55%), *iucC* (54.2%), *iss* (51.7%), and *ompT* (28.3%). Other relevant ExPEC-associated genes detected were *kpsMII* (15.8%), *chuA* (9.2%), *papA* (5.8%), *papC* (5.8%), *afaB* (2.5%), *afaC* (2.5%), and *yfcV* (0.8%). According to two main operational definitions (Johnson et al., 2003; Spurbeck et al., 2012), nine EAEC isolates were classified as presumptive ExPEC, one isolate was classified as UPEC, and two isolates were classified as both ExPEC and UPEC (Supplementary Table 2). Therefore, 10% of EAEC isolates in our collection revealed an additional urinary/systemic pathogenic potential.

Regarding the acquired antibiotic resistance genes profiles, 40 (33.3%) of 120 isolates harbored genes conferring resistance to at least one antibiotic category and 28 (23.3%) harbored genes conferring resistance to three or more categories and were therefore considered multidrug resistant (MDR) based on the WGS prediction (Supplementary Table 2). There were no common resistance determinant profiles and the highest number of isolates that shared the same genotypic resistance determinant profile (*aph(3″)-Ib*/*aph(6)-Id*/*sul2*/*bla*_TEM-1C_) was seven. As for the presence of extended-spectrum beta-lactamases (ESBLs), conferring resistance to third generation cephalosporins, the *bla*_CTX-M-15_ and *bla*_CTX-M-14_ genes were harbored by two and one isolate, respectively (Supplementary Table 2).

## 4. Discussion

We undertook a large prospective study of diarrheal disease at five hospitals located in different Spanish provinces widely distributed geographically, with the purpose of determining the role of EAEC among patients seeking medical care. The study demonstrated that EAEC is frequently detected among patients with diarrhea in Spain (7.8%), especially in children ≤5 years old, among which EAEC prevalence reached 9.8%. This finding corresponds well with previous studies demonstrating a remarkable predisposition to EAEC infection in children ≤5 years of age and suggesting that the prevalence and significance of EAEC infections depend on age (Pabst et al., 2003; Cohen et al., 2005). Although it is possible that some EAEC detected in this study are not pathogenic or represent colonization rather than infection, the presence of other more established bacterial enteric pathogens (e.g., *Salmonella* spp., *Campylobacter* spp.) was ruled out per study protocol, and other clinically relevant DEC pathotypes (STEC, ETEC, and EIEC) were co-detected in only 4.3% of cases of EAEC infection. Furthermore, 90% of our EAEC isolates met the new molecular definition of EAEC comprising *E. coli* strains harboring AggR and its adhesin dependent factors (AAF(I-V) or CS22), recently proposed by Boisen et al. (2020), and could thus be considered as true EAEC. Therefore, EAEC was the only bacterial enteric pathogen detected in a significant proportion of cases of diarrhea, none of which had a history of recent travel abroad, thus suggesting that EAEC is an important domestically acquired bacterium responsible for endemic diarrhea in Spain. This proportion of patients with non-travel related diarrhea who were demonstrated to be infected with EAEC was unexpectedly high, as EAEC prevalence is expected to be higher among travelers from industrialized countries visiting less-developed regions. However, our findings are supported by studies conducted in other high-income countries that also showed relatively frequent detection of EAEC among patients with diarrhea (Supplementary Table 3), with detection rates ranging from 1.9 to 5.9% in the general population (Wilson et al., 2001; Nataro et al., 2006; Hardegen et al., 2010; Tam et al., 2012; Cybulski et al., 2018; Hebbelstrup Jensen et al., 2018) and up to 11.9% in children ≤5 years old (Pabst et al., 2003; Nataro et al., 2006; Tobias et al., 2015). In particular, Pabst et al. (2003) and Cohen et al. (2005) detected EAEC from children ≤5 years with diarrhea significantly more frequently than from healthy children (11.9% vs. 2.2 and 9.2% vs. 3.3%, respectively), although the association of EAEC with diarrhea did not achieve statistical significance in our case–control study. On the contrary, as expected, our detection rates are substantially lower than those generally reported in developing countries, with EAEC prevalences up to 39% in this setting (Gonzalez et al., 1997; Okeke et al., 2000; Modgil et al., 2021; Manhique-Coutinho et al., 2022).

**Figure 2:**
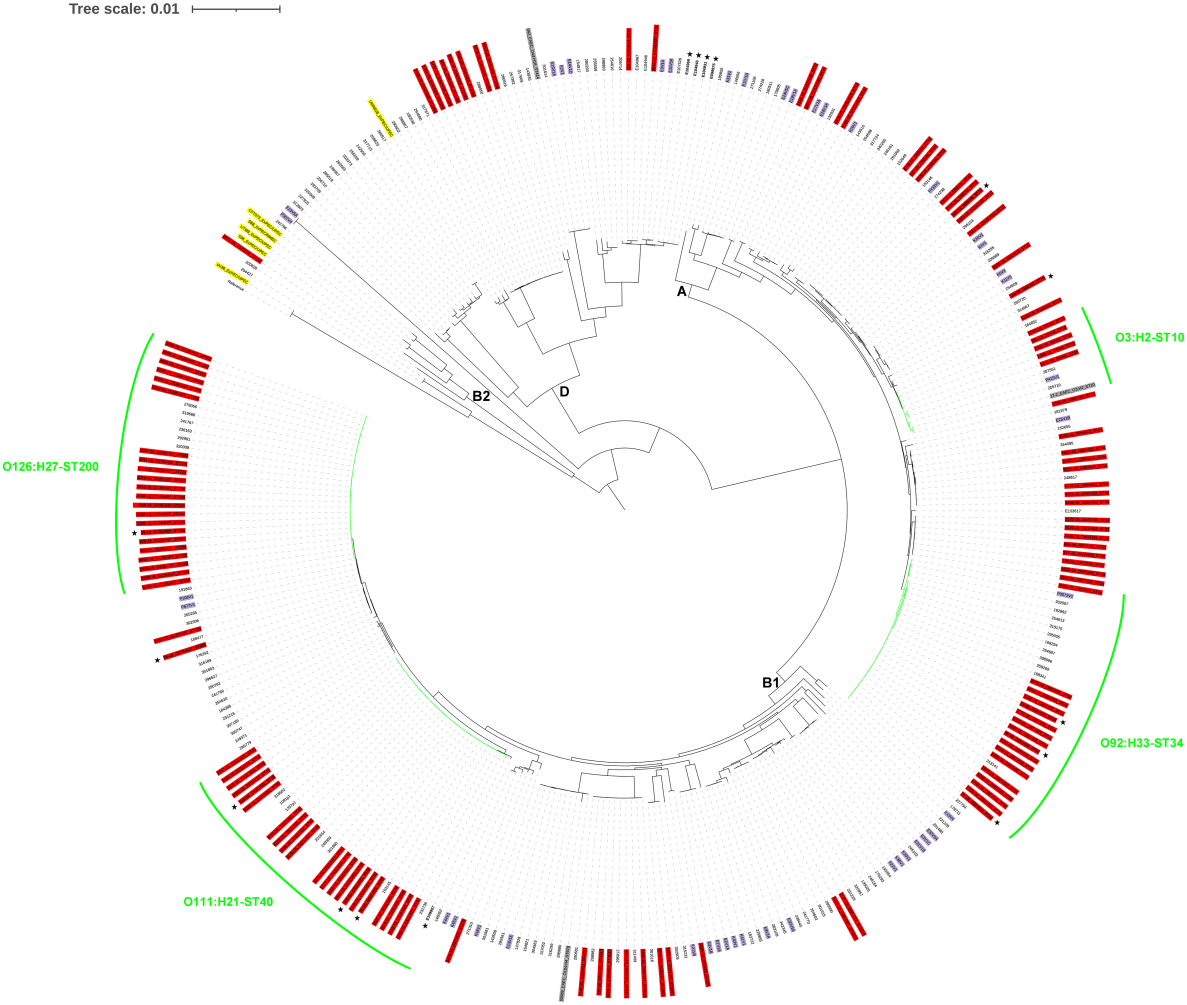
Phylogenomic analysis of the enteroaggregative *Escherichia coli* genomes. The whole-genome phylogeny was constructed from 690,639 conserved SNP sites per genome that were identified compared against the reference genome of the *E. coli* strain IAI39 (GenBank accession no. NC_011750.1). The isolates from Spain (this study) are colored in red, those from Egypt, Kenya, or Peru are colored in purple, and those from the United Kingdom are not colored. EAEC and ExPEC reference genomes are colored in gray and yellow, respectively. Isolates obtained from asymptomatic controls are in bold and indicated by a star in the outer ring of labels. The most important serogenotype-ST combinations identified in this study are highlighted in green. The *E. coli* phylogroups are designated by letters (A, B1, B2, and D) on the interior of the phylogeny based on the inclusion of both EAEC isolates sequenced in this study and reference strains. The tree scale indicates the distance of 0.01 nucleotide changes per site.

Although there were 26 different serogenotypes among the 120 isolates, 82% were restricted to only nine serogenotypes, very homogeneous with respect to ST, with some particularly common serogenotype-ST combinations, such as O126:H27-ST200, O111:H21-ST40, and O92:H33-ST34, comprising 50% of isolates. This finding is in contrast to previous studies reporting a higher diversity in terms of serotyping and MLST among EAEC clinical isolates from the United States or the United Kingdom (Do Nascimento et al., 2017; Beczkiewicz et al., 2019), probably because such studies did not rule out travel-related infections. These same serotypes are among the most frequently reported in EAEC strains from other high-income countries (Shazberg et al., 2003; Tobias et al., 2015; Hebbelstrup Jensen et al., 2016; Imuta et al., 2016; Do Nascimento et al., 2017; Beczkiewicz et al., 2019), and even linked to outbreaks of gastroenteritis (Yatsuyanagi et al., 2002; Harada et al., 2007; Scavia et al., 2008; Dallman et al., 2012), although rarely identified among EAEC strains from developing countries (Boisen et al., 2020; Petro et al., 2020). Indeed, in the whole-genome phylogeny including 324 EAEC genomes from developing (Egypt, Kenya, and Peru) and high-income (Spain and the United Kingdom) countries (Figure 2), isolates of the most common serogenotype-ST combinations clustered together in independent groups consisting exclusively (O126:H27-ST200 and O111:H21-ST40) or almost exclusively (O92:H33-ST34) of isolates originating from high-income countries. Moreover, as revealed in the whole-genome phylogenies specific for each of these subtypes (Supplementary Figures 1–3), isolates from the United Kingdom were interleaved with those from Spain belonging to the same serogenotype-ST combination. As isolates in the present study were not travel-related, this suggests that O126:H27-ST200, O111:H21-ST40, and O92:H33-ST34 are the most important domestically acquired EAEC subtypes in Spain, and probably also in other high-income countries. In particular, O111:H21-ST40 strains have been recently proposed to have a higher intrinsic potential to cause diarrheal disease in the United Kingdom (Ellis et al., 2020). Nevertheless, we found O111:H21-ST40, and all the aforementioned common EAEC subtypes, both in isolates obtained from patients and those from asymptomatic controls. Indeed, most of the isolates originating from asymptomatic carriage in the present study showed combinations of phylogroup, serogenotype, ST, virulence genes, and antibiotic resistance genes already found among clinical isolates (Supplementary Table 2). This similarity between isolates from cases and controls was also revealed in the O126:H27-ST200, O111:H21-ST40, and O92:H33-ST34 specific phylogenies (Supplementary Figures 1–3), in which isolates from controls were interleaved with those from cases belonging to the same serogenotype-ST combination. Although these findings could be influenced by the scarce number of isolates obtained from asymptomatic controls in this study (n = 10), they suggest that the same EAEC strains infected both patients and asymptomatic individuals. This is in contrast to the hypothesis that EAEC strains isolated from patients with diarrhea would belong to different subtypes and/or harbor putative virulence factors distinct from, or more commonly than, those isolated from asymptomatic controls (Boisen et al., 2012, 2020).

The molecular characterization of isolates together with their origin and collection date could suggest possible unnoticed episodes of transmission of the most important domestically acquired EAEC subtypes (Supplementary Table 2) and this could be assessed from the whole-genome phylogenies specific for each of these subtypes. According to criteria proposed by Pightling et al. (2018) for interpreting WGS analyses of foodborne bacteria for outbreak investigations, monophyletic groups of *E. coli* isolates with a median pairwise distance of 20 or fewer SNPs, a bootstrap support of 90 or higher, and some epidemiological evidence support transmission episodes. In this study, such analyses revealed four possible episodes of EAEC O126:H27-ST200 transmission involving 2–6 patients (Supplementary Figure 1), one possible episode of EAEC O111:H21-ST40 transmission involving two patients (Supplementary Figure 2), and two possible episodes of EAEC O92:H33-ST34 transmission involving 2–3 patients (Supplementary Figure 3).

AAF/V was the most prevalent variant in our collection, as previously reported in Denmark (Hebbelstrup Jensen et al., 2017), with 70% of our AAF/V-harboring isolates belonging to the predominant EAEC subtype O111:H21-ST40 and thus explaining its predominance in our setting. Likewise, 92% of our AAF/II-harboring isolates and 68% of our AAF/I-harboring isolates belonged to the predominant EAEC subtypes O126:H27-ST200 and O92:H33-ST34, respectively, also explaining their predominance in our setting. Of particular interest is the frequent detection of AAF/IV in our isolates, all of them lacking *aar* and harboring *sepA*, as such strains have been recently proposed to be more diarrheagenic than other EAEC (Boisen et al., 2020; Petro et al., 2020). However, certain AAF/IV-harboring isolates in our study showed what appears to be a novel AAF/IV fimbrial cluster where the minor pilin subunit gene *agg4B* has been replaced by *afaD*. In particular, this apparently new organization of AAF/IV was found in all O55:H21-ST4213 isolates (*n* = 5), all O44:H18-ST2959 isolates (*n* = 2), all O121:H27-ST1891 isolates (*n* = 2), and some O99:H4-ST10 isolates (*n* = 3). This finding was also confirmed in five O55:H21-ST4213 isolates and one O44:H18-ST2959 isolate originating from the United Kingdom (Do Nascimento et al., 2017; Supplementary Table 4), thus supporting the idea that the epidemiological scenario of endemic EAEC infections would be very similar in different industrialized countries. The identification and characterization of the genetic environment of this apparently novel AAF/IV fimbrial cluster warrants further investigation. Notably, one isolate was found to harbor the genes for both AAF/I and AAF/V, a phenomenon described previously only for AAF/III and AAF/V (Jønsson et al., 2017; Petro et al., 2020). Again, the identification and characterization of the genetic environment of both AAF variants in this particular EAEC isolate warrants further investigation. One of the isolates without a known AAF variant harbored the *cseA* gene, indicative of the presence of the non-fimbrial ETEC colonization factor CS22 (Pichel et al., 2000), recently identified in strains lacking an identifiable AAF but harboring different putative EAEC virulence factors and being considered typical EAEC by genomic criteria (Boisen et al., 2020; Petro et al., 2020). This *cseA*-positive isolate belonged to O9:H21-ST155, which has been recently identified among CS22-like harboring EAEC strains originating from Kenya (Petro et al., 2020) and Mozambique (Boisen et al., 2020).

Apart from its role as an enteric pathogen, EAEC has emerged as a causative agent of urinary tract infection (UTI) and bacteremia in the last years (Boll et al., 2020; Mandomando et al., 2020). In particular, phylogroup A and AAF/I have been associated with uropathogenicity and AAF/V with bacteremia (Nunes et al., 2017; Mandomando et al., 2020). In the present study, 11 EAEC isolates were classified as presumptive ExPEC and four of them specifically belonged to serotype O3:H2, phylogroup A, and ST10 and harbored AAF/I. Additionally, we analyzed four previously sequenced EAEC O3:H2-ST10 genomes, including the EAEC reference strain 17–2, and three of them were also classified as presumptive ExPEC (Supplementary Table 5). It should be noted that EAEC O3:H2-ST10 isolates classified as ExPEC consistently harbored AAF/I (with the only exception of one isolate harboring AAF/V), whereas isolates not classified as ExPEC harbored AAF/III and clustered together, far from the ExPEC isolates harboring AAF/I and AAF/V, in the specific phylogeny (Supplementary Figure 4), thus suggesting the importance of AAF/I in extraintestinal EAEC infections. Although the EAEC prototype strain 17–2 had been previously proposed to present some ExPEC/UPEC characteristics (Gomes et al., 1995; Schüroff et al., 2021), to the best of our knowledge, this is the first study to reveal that phylogroup A EAEC O3:H2-ST10 harboring AAF/I is a clonal lineage of EAEC that could be specifically associated with extraintestinal infections. Furthermore, one of the two EAEC isolates classified as ExPEC/UPEC belonged to serotype O153:H4, phylogroup B2, and ST131 and harbored AAF/V and *fimH27* (data not shown). It was the only EAEC isolate that belonged to phylogroup B2 in our collection and clustered together with ExPEC/UPEC reference strains belonging to phylogroup B2 but far from the rest of diarrheagenic EAEC isolates and reference strains (Figure 2), thus supporting the idea that authentic enteric and urinary/systemic pathogens can be found among strains meeting the definition of EAEC (Boisen et al., 2020). Indeed, the ST131 *H27* sublineage is a novel subclone of *E. coli* ST131 that has acquired the EAEC diarrheagenic phenotype, spread across multiple continents, and caused multiple outbreaks of community-acquired bacteremia and recurrent UTIs (Boll et al., 2020; Mandomando et al., 2020).

Multidrug-resistance defined as antibiotic resistance to at least three antibiotic categories (Magiorakos et al., 2012) is widespread among foodborne and waterborne enteric pathogens, including EAEC (Hebbelstrup Jensen et al., 2014, 2016; Do Nascimento et al., 2017; Beczkiewicz et al., 2019; Boisen et al., 2020). In our study, 23.3% of the EAEC isolates were considered MDR based on the WGS prediction, and the majority of them originated from children ≤5 years old. As expected, this level of MDR was much lower than that detected in previous studies conducted in developing countries, with MDR detection rates close to 80% (Boisen et al., 2020). However, this finding is in contrast to previous studies also based on the WGS prediction of antibiotic resistance and conducted in high-income countries like the United Kingdom and the United States (Do Nascimento et al., 2017; Beczkiewicz et al., 2019), with MDR detection rates of 56.8 and 51.6%, respectively, again probably because such studies did not rule out travel-associated infections. Of special concern are the abundant ESBL production and the increased resistance to quinolones in EAEC strains (Herrera-León et al., 2015; Imuta et al., 2016; Guiral et al., 2019). In particular, the presence of CTX-M ESBL variants (*bla*_CTX-M-15_ and *bla*_CTX-M-14_ genes) was detected only in 2.5% of EAEC isolates obtained from cases of endemic diarrhea in Spain. Again, this finding is in contrast to the 20% detected among EAEC isolates from patients with gastrointestinal symptoms in the United Kingdom, and this is probably due to the extremely high percentage of patients reporting travel abroad within 7 days of onset of symptoms in that study (Do Nascimento et al., 2017). While treatment of EAEC infection is not based on antibiotics in the majority of cases, as many EAEC infections are self-limited, evaluating antibiotic susceptibility is important in cases where antibiotic use is clinically indicated.

Our study had several strengths. It is the first Spanish study to explore the role of EAEC in endemic diarrhea and one of the largest studies conducted in an industrialized country to date. As samples were collected from five provinces widely distributed geographically, our results might be representative of the whole country. Unlike most previous studies, we ruled out travel-related diarrheal episodes and those in which other bacterial pathogens were present. We generated one of the most complete characterizations of EAEC strains associated with illness acquired in industrialized countries to date. However, it also had several limitations. Detection of EAEC was based on PCR amplification of a well-known EAEC target but functional testing using the Hep-2 adherence assay was not performed. Although the adherence test remains the “gold standard” for diagnosing EAEC infection, it is resource intensive and requires strict adherence to protocol and specialized facilities. This issue could have underestimated the EAEC prevalence in our study and could be especially significant for isolates negative for both AAF and CS22 not meeting the new molecular definition of EAEC. It was not possible to elucidate the exact etiology of the disease outcome, as samples were not tested for the presence of *Clostridioides difficile* toxins, parasites, or viruses. The scarce number of control subjects fulfilling the inclusion criteria may have compromised the accuracy of some of our results. We did not collect comprehensive data on symptoms, treatments, outcomes, or risk factors. Finally, phenotypic resistance profile information was not available as conventional antimicrobial susceptibility testing was not performed.

## 5. Concluding remarks

EAEC was the only bacterial enteric pathogen detected in a significant proportion of cases of endemic diarrhea in Spain, especially in children ≤5 years old. In particular, O126:H27-ST200, O111:H21-ST40, and O92:H33-ST34 were the most important subtypes, with all of them infecting both patients and asymptomatic individuals. A subset of these domestically acquired EAEC strains were additionally classified as ExPEC, UPEC, or both, and belonged to clonal lineages that could be specifically associated with extraintestinal infections, thus revealing an additional urinary/systemic pathogenic potential. These data highlight the convenience of routinely testing for EAEC especially for children ≤5 years old with diarrheal disease and those patients in which no other pathogen can be identified.

## Data availability statement

The datasets presented in this study can be found in online repositories. The names of the repository/repositories and accession number(s) can be found in the article/Supplementary material.

## Ethics statement

The studies involving human participants were reviewed and approved by Comisión Central de Investigación, Gerencia Asistencial de Atención Primaria, Servicio Madrileño de Salud. Written informed consent to participate in this study was provided by the participants’ legal guardian/next of kin.

## Author contributions

SS and RE conceived and designed the study. Material preparation, data collection and analysis were performed by MR, RM-R, FG-S, MF, ME, IO, RR, and ML. The first draft of the manuscript was written by SS and all authors commented on previous versions of the manuscript. All authors contributed to the article and approved the submitted version.

## Funding

This work was supported by Institute of Health Carlos III, Spanish Ministry of Economy and Competitiveness (MPY-1042/14, PI14CIII/00051, and PI18CIII/00043) and the SHARP Joint Action (2019 – March 2023) co-funded by the Health Programme of the European Union. SS performed this work while under a research contract from the Miguel Servet program from the Spanish Ministry of Economy and Competitiveness (CP13/00237).

## Conflict of interest

The authors declare that the research was conducted in the absence of any commercial or financial relationships that could be construed as a potential conflict of interest.

## Publisher’s note

All claims expressed in this article are solely those of the authors and do not necessarily represent those of their affiliated organizations, or those of the publisher, the editors and the reviewers. Any product that may be evaluated in this article, or claim that may be made by its manufacturer, is not guaranteed or endorsed by the publisher.

## Footnotes

^1^ https://cge.cbs.dtu.dk//services

^2^ http://clermontyping.iame-research.center

^3^ http://github.com/tseemann/snippy
