# Supplementary material for: Enteroaggregative Escherichia coli as etiological agent of endemic diarrhea in Spain: A prospective multicenter prevalence study with molecular characterization of isolates
Source: Front Microbiol. 2023 Mar 20;14:1120285. doi: 10.3389/fmicb.2023.1120285 (PMC10100739; doi:10.3389/fmicb.2023.1120285)
Supplement: Supplementary file 4 [file Data_Sheet_1.docx]

Supplementary Material

Enteroaggregative Escherichia coli as etiological agent of endemic diarrhea in Spain: a prospective multicenter prevalence study with molecular characterization of isolates

María Teresa Llorente^1,2^, Raquel Escudero^2^, Raquel Ramiro^1^, María Antonia Remacha^3^, Rocío Martínez-Ruiz^4^, Fátima Galán-Sánchez^5^, Mónica de Frutos^6^, Matilde Elía^7^, Isabel Onrubia^8^, Sergio Sánchez^1,*^

^1^Reference and Research Laboratory on Food and Waterborne Bacterial Infections, National Center for Microbiology, Institute of Health Carlos III, Majadahonda, Madrid, Spain

^2^Reference and Research Laboratory on Special Pathogens, National Center for Microbiology, Institute of Health Carlos III, Majadahonda, Madrid, Spain

^3^Complejo Asistencial Universitario de León, León, Spain

^4^Hospital Puerta de Hierro Majadahonda, Majadahonda, Spain

^5^Hospital Universitario Puerta del Mar, Cádiz, Spain

^6^Hospital Universitario del Río Hortega, Valladolid, Spain

^7^Hospital Universitario de Navarra, Pamplona, Spain

^8^Centro de Salud Valle de la Oliva, Majadahonda, Spain

*** Correspondence:** Dr. Sergio Sánchez Prieto: sergio.sanchez@isciii.es

# Supplementary Tables

**Supplementary Table 1.** List of the *Escherichia coli* reference genomes included in the phylogenomic analyses.

| Strain | Pathotype | Phylogroup | Bioproject ID | Accession number |
| --- | --- | --- | --- | --- |
| 55989 | EAEC | B1 | PRJNA33413 | SAMEA3138229 |
| 042 | EAEC | D | PRJEA40647 | SAMEA2272277 |
| 17-2 | EAEC | A | PRJNA646864 | SAMN15567421 |
| 536 | ExPEC/UPEC | B2 | PRJNA16235 | SAMN02604181 |
| S88 | ExPEC/NMEC | B2 | PRJNA33375 | SAMEA3138231 |
| UTI89 | ExPEC/UPEC | B2 | PRJNA16259 | SAMN00000110 |
| CFT073 | ExPEC/UPEC | B2 | PRJNA313 | SAMN02604094 |
| UMN026 | ExPEC/UPEC | D | PRJNA33415 | SAMEA3138233 |
| IAI39 | ExPEC/UPEC | F | PRJNA33411 | SAMEA3138234 |
| Abbreviations: EAEC, enteroaggregative *E. coli*; ExPEC, extraintestinal pathogenic *E. coli*; uropathogenic *E. coli*; NMEC, neonatal meningitis *E. coli* | | | | |

**Supplementary Table 2.** Description and genomic characteristics of the enteroaggregative *Escherichia coli* isolates obtained from patients with endemic diarrhea (cases) and asymptomatic individuals (controls).

**Supplementary Table 3.** Main enteroaggregative *Escherichia coli* prevalence studies conducted in high-income countries with PCR-based methods.

| Year | Country | | Study population | No. of patients (% EAEC positive) | Association with diarrhea | Reference |
| --- | --- | --- | --- | --- | --- | --- |
| 1997 | Germany | | Children <16 years old | 798 cases (2.0), 580 controls (0) | Yes | (Huppertz et al., 1997) |
| 1999 | UK | | General population | 3,506 cases (4.6), 2,772 controls (1.7) | Yes | (Wilson et al., 2001) |
| 2003 | Switzerland | | Children ≤5 years old | 151 cases (11.9), 91 controls (2.2) | Yes | (Pabst et al., 2003) |
|  |  | | Children >5 years old | 36 cases (2.8), 46 controls (2.2) |  |  |
| 2005 | USA | | Children <1 year old | 343 inpatient cases (4.7), 362 emergency department cases (10.0), 363 controls (1.4) | Yes | (Cohen et al., 2005) |
|  |  | | Children ≤5 years old | 563 inpatient cases (2.2), 684 emergency department cases (9.2), 486 controls (3.3) | No |  |
| 2006 | USA | | General population | 823 cases (4.5), 412 controls (1.7) | Yes | (Nataro et al., 2006) |
|  |  | | Children ≤5 years old | 317 cases (5.0), 56 controls (1.8) |  |  |
| 2009 | USA | | Adults | 253 cases (7.0), 751 controls (3.0) | Yes | (Cennimo et al., 2009) |
| 2010 | Germany | | General population | 1610 (4.8) | NA | (Hardegen et al., 2010) |
| 2012 | USA | | Children | 254 cases (3.1), 452 controls (0.9) | Yes | (Denno et al., 2012) |
| 2012 | UK | | General population | 874 community cases (1.4), 782 cases presenting to healthcare (1.9) | NA | (Tam et al., 2012) |
| 2015 | Israel | | Children <5 years old | 307 (8.8) | NA | (Tobias et al., 2015) |
| 2015 | USA | | Children <5 years old | 165 (2.4) | NA | (Foster et al., 2015) |
| 2017 | USA | | Children <5 years old | 511 (2.9) | NA | (Stockmann et al., 2017) |
|  |  | | Children 5-17 years old | 578 (2.6) |  |  |
| 2018 | USA | | Children | 857 cases (2.0), 410 controls (4.0) | No | (Imdad et al., 2018) |
| 2018 | Italy | | Children <5 years old | 1,186 (6.2) | NA | (Calderaro et al., 2018) |
|  |  | | Children 5-14 years old | 530 (5.1) |  |  |
| 2018 | USA | | General population | 1,887 (5.9) | NA | (Cybulski et al., 2018) |
| 2018 | Denmark | | General population | 10,036 (4.6) | NA | (Hebbelstrup Jensen et al., 2018) |
| Abbreviations: EAEC, enteroaggregative *E. coli*; NA, not applicable | | | | | | |
|  | |  |  |  |  |  |

**References in Supplementary Table 3**

Calderaro, A., Martinelli, M., Buttrini, M., Montecchini, S., Covan, S., Rossi, S., et al. (2018). Contribution of the FilmArray Gastrointestinal Panel in the laboratory diagnosis of gastroenteritis in a cohort of children: a two-year prospective study. *Int J Med Microbiol* 308(5), 514-521. doi: 10.1016/j.ijmm.2018.04.007.

Cennimo, D., Abbas, A., Huang, D. B., and Chiang, T. (2009). The prevalence and virulence characteristics of enteroaggregative *Escherichia coli* at an urgent-care clinic in the USA: a case-control study. *J Med Microbiol* 58(Pt 4), 403-407. doi: 10.1099/jmm.0.005793-0.

Cohen, M. B., Nataro, J. P., Bernstein, D. I., Hawkins, J., Roberts, N., and Staat, M. A. (2005). Prevalence of diarrheagenic *Escherichia coli* in acute childhood enteritis: a prospective controlled study. *J Pediatr* 146(1), 54-61. doi: 10.1016/j.jpeds.2004.08.059.

Cybulski, R. J., Jr., Bateman, A. C., Bourassa, L., Bryan, A., Beail, B., Matsumoto, J., et al. (2018). Clinical impact of a multiplex gastrointestinal polymerase chain reaction panel in patients with acute gastroenteritis. *Clin Infect Dis* 67(11), 1688-1696. doi: 10.1093/cid/ciy357.

Denno, D. M., Shaikh, N., Stapp, J. R., Qin, X., Hutter, C. M., Hoffman, V., et al. (2012). Diarrhea etiology in a pediatric emergency department: a case control study. *Clin Infect Dis* 55(7), 897-904. doi: 10.1093/cid/cis553.

Foster, M. A., Iqbal, J., Zhang, C., McHenry, R., Cleveland, B. E., Romero-Herazo, Y., et al. (2015). Enteropathogenic and enteroaggregative *E. coli* in stools of children with acute gastroenteritis in Davidson County, Tennessee. *Diagn Microbiol Infect Dis* 83(3), 319-324. doi: 10.1016/j.diagmicrobio.2015.07.016.

Hardegen, C., Messler, S., Henrich, B., Pfeffer, K., Wurthner, J., and MacKenzie, C. R. (2010). A set of novel multiplex Taqman real-time PCRs for the detection of diarrhoeagenic *Escherichia coli* and its use in determining the prevalence of EPEC and EAEC in a university hospital. *Ann Clin Microbiol Antimicrob* 9, 5. doi: 10.1186/1476-0711-9-5.

Hebbelstrup Jensen, B., Adler Sørensen, C., Hebbelstrup Rye Rasmussen, S., Rejkjær Holm, D., Friis-Møller, A., Engberg, J., et al. (2018). Characterization of diarrheagenic enteroaggregative *Escherichia coli* in Danish adults—Antibiotic treatment does not reduce duration of diarrhea. *Front Cell Infect Microbiol* 8, 306. doi: 10.3389/fcimb.2018.00306.

Huppertz, H. I., Rutkowski, S., Aleksic, S., and Karch, H. (1997). Acute and chronic diarrhoea and abdominal colic associated with enteroaggregative *Escherichia coli* in young children living in western Europe. *Lancet* 349(9066), 1660-1662. doi: 10.1016/S0140-6736(96)12485-5.

Imdad, A., Foster, M. A., Iqbal, J., Fonnesbeck, C., Payne, D. C., Zhang, C., et al. (2018). Diarrheagenic *Escherichia coli* and acute gastroenteritis in children in Davidson County, Tennessee, United States: a case-control study. *Pediatr Infect Dis J* 37(6), 543-548. doi: 10.1097/INF.0000000000001908.

Nataro, J. P., Mai, V., Johnson, J., Blackwelder, W. C., Heimer, R., Tirrell, S., et al. (2006). Diarrheagenic *Escherichia coli* infection in Baltimore, Maryland, and New Haven, Connecticut. *Clin Infect Dis* 43(4), 402-407. doi: 10.1086/505867.

Pabst, W. L., Altwegg, M., Kind, C., Mirjanic, S., Hardegger, D., and Nadal, D. (2003). Prevalence of enteroaggregative *Escherichia coli* among children with and without diarrhea in Switzerland. *J Clin Microbiol* 41(6), 2289-2293. doi: 10.1128/jcm.41.6.2289-2293.2003.

Stockmann, C., Pavia, A. T., Graham, B., Vaughn, M., Crisp, R., Poritz, M. A., et al. (2017). Detection of 23 gastrointestinal pathogens among children who present with diarrhea. *J Pediatric Infect Dis Soc* 6(3), 231-238. doi: 10.1093/jpids/piw020.

Tam, C. C., O'Brien, S. J., Tompkins, D. S., Bolton, F. J., Berry, L., Dodds, J., et al. (2012). Changes in causes of acute gastroenteritis in the United Kingdom over 15 years: microbiologic findings from 2 prospective, population-based studies of infectious intestinal disease. *Clin Infect Dis* 54(9), 1275-1286. doi: 10.1093/cid/cis028.

Tobias, J., Kassem, E., Rubinstein, U., Bialik, A., Vutukuru, S. R., Navaro, A., et al. (2015). Involvement of main diarrheagenic *Escherichia coli*, with emphasis on enteroaggregative *E. coli*, in severe non-epidemic pediatric diarrhea in a high-income country. *BMC Infect Dis* 15, 79. doi: 10.1186/s12879-015-0804-4.

Wilson, A., Evans, J., Chart, H., Cheasty, T., Wheeler, J. G., Tompkins, D., et al. (2001). Characterisation of strains of enteroaggregative *Escherichia coli* isolated during the infectious intestinal disease study in England. *Eur J Epidemiol* 17(12), 1125-1130. doi: 10.1023/a:1021224915322.

**Supplementary Table 4.** Genomic characteristics of AAF/IV-harboring enteroaggregative *Escherichia coli* strains originating from the UK.

**Supplementary Table 5.** Genomic characteristics of the O3:H2-ST10 enteroaggregative *Escherichia coli* strains included in the phylogenomic analyses.

# Supplementary Figures

**Supplementary Figure 1. Phylogenomic analysis of the O126:H27-ST200 enteroaggregative *Escherichia coli* genomes.** A SNP matrix was generated for 28 isolates with CSI Phylogeny 1.4 (https://cge.food.dtu.dk/services/CSIPhylogeny) and the published genome of *E. coli* strain A41 (GenBank accession no. NZ_CP028735.1) as a reference, according to KmerFinder 3.2 results. The SNP matrix was phylogenetically analyzed with RAxML 8.2.12 (Stamatakis, 2014) with a GTR model. Branch labels indicate support values for 1000 bootstrap replicates. Bootstrap values less than 90 are not shown. Isolates obtained from asymptomatic controls are in bold and indicated by a star. Monophyletic groups of isolates with a median pairwise distance of 20 or fewer SNPs, a bootstrap support of 90 or higher, and some epidemiological evidence supporting episodes of EAEC transmission (Pightling et al., 2018) are coloured in green. The tree scale indicates the distance of 0.001 nucleotide changes per site.

**Supplementary Figure 2. Phylogenomic analysis of the O111:H21-ST40 enteroaggregative *Escherichia coli* genomes.** A SNP matrix was generated for 33 isolates with CSI Phylogeny 1.4 (https://cge.food.dtu.dk/services/CSIPhylogeny) and the published genome of *E. coli* strain ESBL 15 (GenBank accession no. NZ_CP041678.1) as a reference, according to KmerFinder 3.2 results. The SNP matrix was phylogenetically analyzed with RAxML 8.2.12 (Stamatakis, 2014) with a GTR model. Branch labels indicate support values for 1000 bootstrap replicates. Bootstrap values less than 90 are not shown. Isolates obtained from asymptomatic controls are in bold and indicated by a star. Monophyletic groups of isolates with a median pairwise distance of 20 or fewer SNPs, a bootstrap support of 90 or higher, and some epidemiological evidence supporting episodes of EAEC transmission (Pightling et al., 2018) are coloured in green. The tree scale indicates the distance of 0.001 nucleotide changes per site.

**Supplementary Figure 3. Phylogenomic analysis of the O92:H33-ST34 enteroaggregative *Escherichia coli* genomes.** A SNP matrix was generated for 27 isolates with CSI Phylogeny 1.4 (https://cge.food.dtu.dk/services/CSIPhylogeny) and the published genome of *E. coli* strain BR1220 (GenBank accession no. NZ_CP093068.1) as a reference, according to KmerFinder 3.2 results. The SNP matrix was phylogenetically analyzed with RAxML 8.2.12 (Stamatakis, 2014) with a GTR model. Branch labels indicate support values for 1000 bootstrap replicates. Bootstrap values less than 90 are not shown. Isolates obtained from asymptomatic controls are in bold and indicated by a star. Monophyletic groups of isolates with a median pairwise distance of 20 or fewer SNPs, a bootstrap support of 90 or higher, and some epidemiological evidence supporting episodes of EAEC transmission (Pightling et al., 2018) are coloured in green. The tree scale indicates the distance of 0.01 nucleotide changes per site.

**Supplementary Figure 4. Phylogenomic analysis of the O3:H2-ST10 enteroaggregative *Escherichia coli* genomes.** A SNP matrix was generated for 9 isolates with CSI Phylogeny 1.4 (https://cge.food.dtu.dk/services/CSIPhylogeny) and the published genome of *E. coli* strain H3 (GenBank accession no. NZ_CP028732.1) as a reference, according to KmerFinder 3.2 results. The SNP matrix was phylogenetically analyzed with RAxML 8.2.12 (Stamatakis, 2014) with a GTR model. Branch labels indicate support values for 1000 bootstrap replicates. Bootstrap values less than 90 are not shown. Isolates obtained from asymptomatic controls are in bold and indicated by a star. Monophyletic groups of isolates with a median pairwise distance of 20 or fewer SNPs, a bootstrap support of 90 or higher, and some epidemiological evidence supporting episodes of EAEC transmission (Pightling et al., 2018) are coloured in green. The tree scale indicates the distance of 0.01 nucleotide changes per site.

**References in Supplementary Figures 1-4**

Stamatakis, A. (2014). RAxML version 8: a tool for phylogenetic analysis and post-analysis of large phylogenies. *Bioinformatics* 30(9), 1312-1313. doi: 10.1093/bioinformatics/btu033.

Pightling, A. W., Pettengill, J. B., Luo, Y., Baugher, J. D., Rand, H., and Strain, E. (2018). Interpreting Whole-Genome Sequence Analyses of Foodborne Bacteria for Regulatory Applications and Outbreak Investigations. *Front Microbiol* 9, 1482. doi: 10.3389/fmicb.2018.01482.
