# Supplementary figures and images for: Enteroaggregative Escherichia coli as etiological agent of endemic diarrhea in Spain: A prospective multicenter prevalence study with molecular characterization of isolates
Source: Front Microbiol. 2023 Mar 20;14:1120285. doi: 10.3389/fmicb.2023.1120285 (PMC10100739; doi:10.3389/fmicb.2023.1120285)

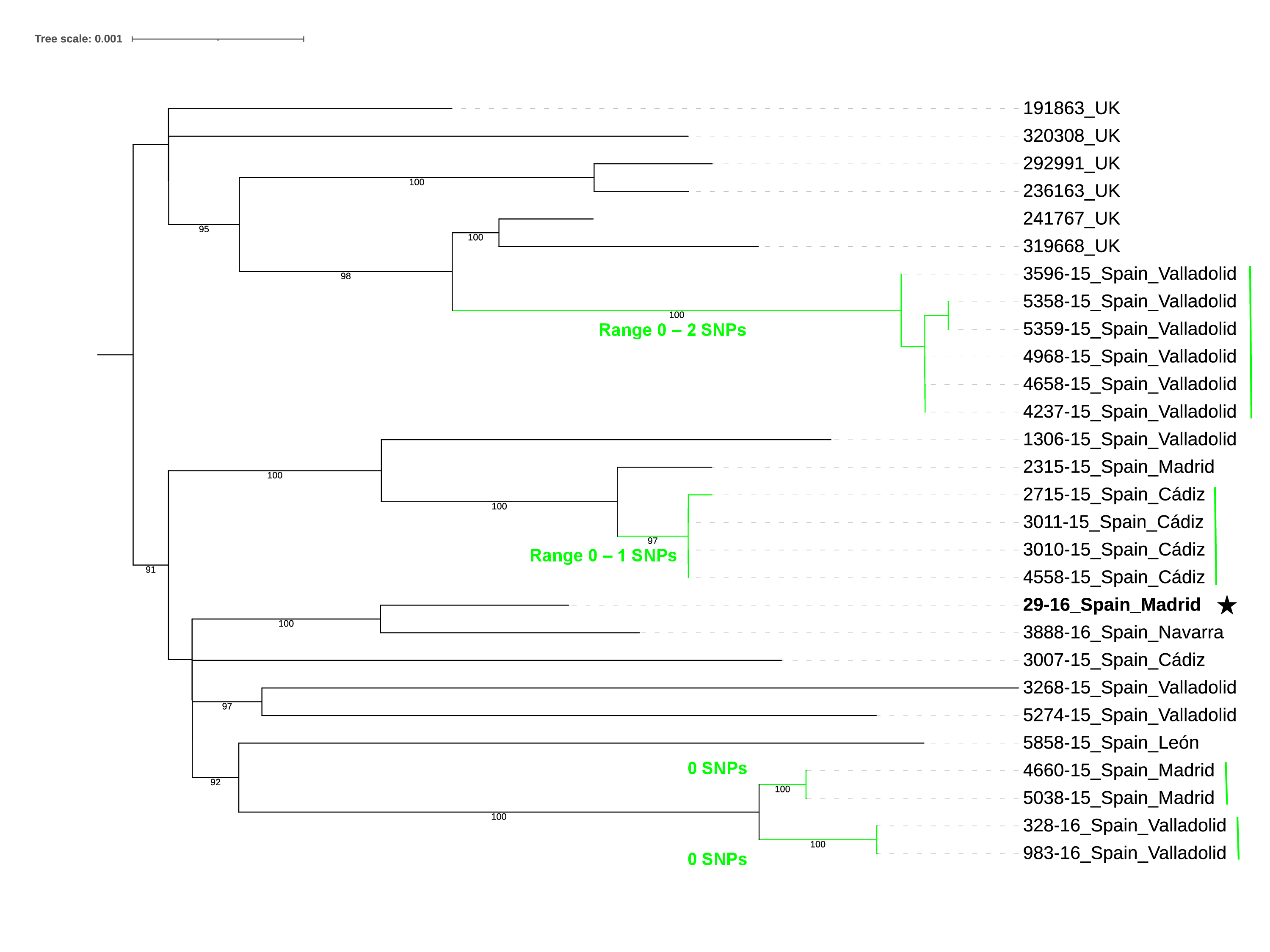

Supplement: Supplementary file 5 [file Image_1.TIFF]

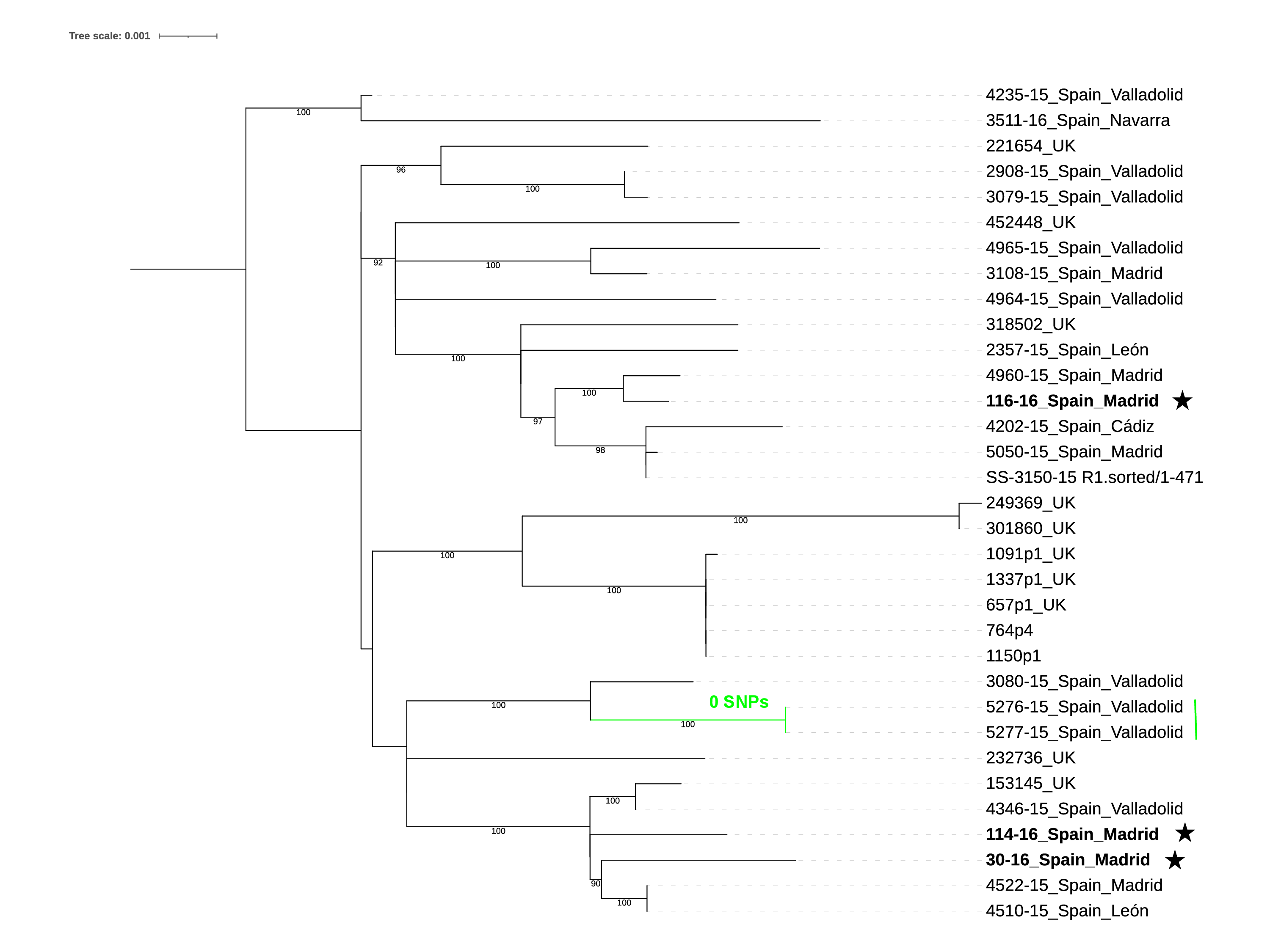

Supplement: Supplementary file 6 [file Image_2.TIFF]

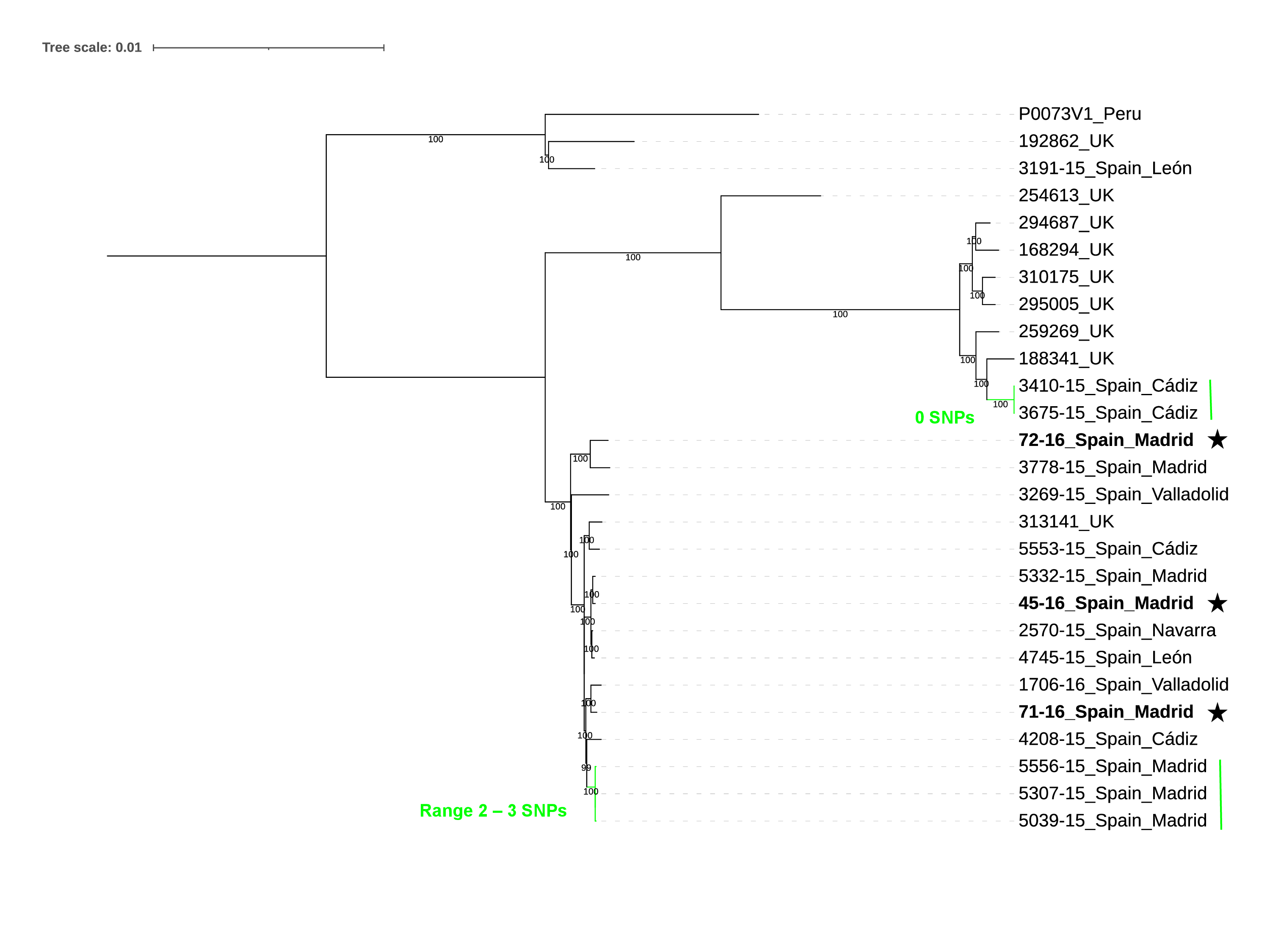

Supplement: Supplementary file 7 [file Image_3.TIFF]

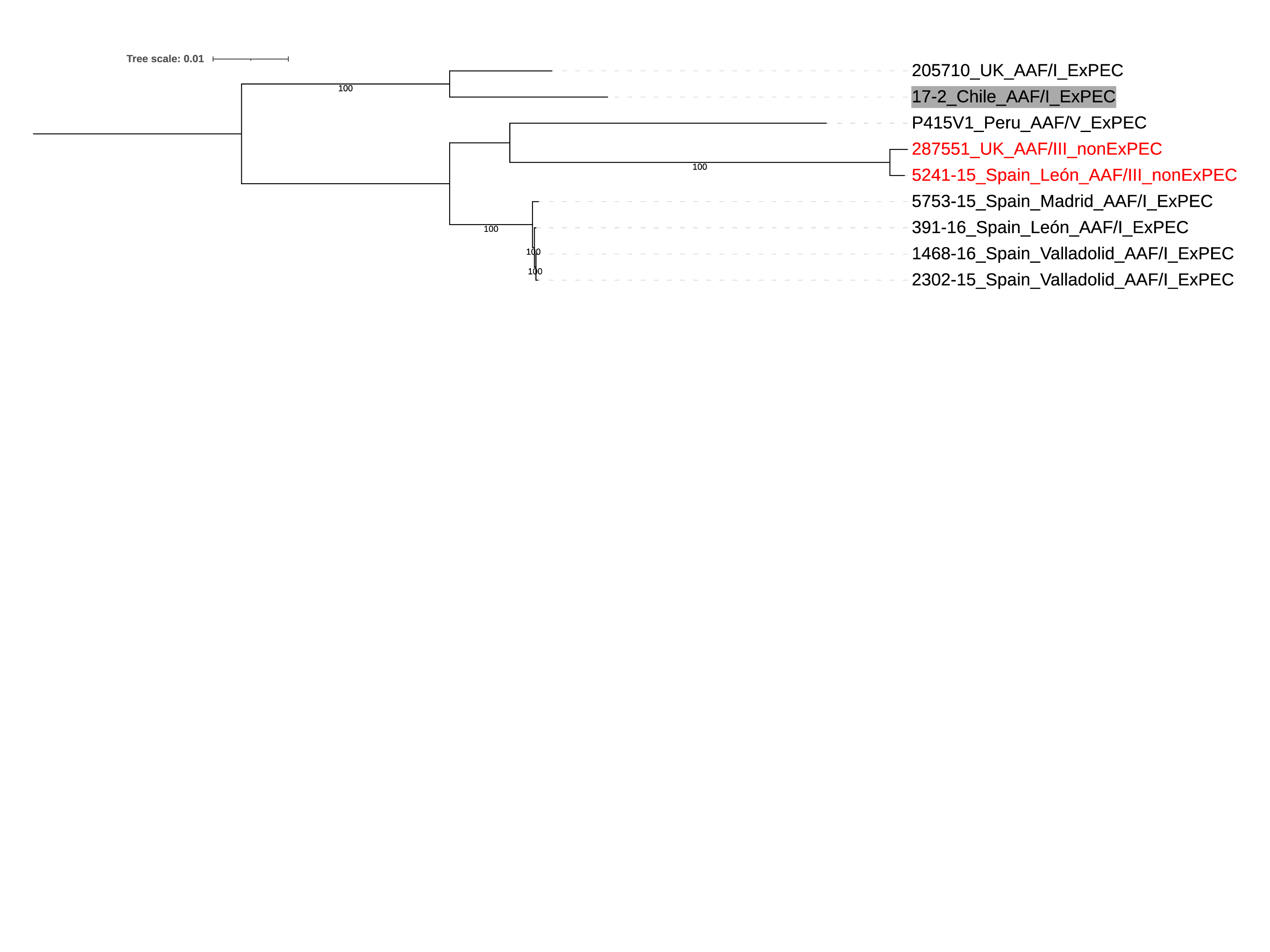

Supplement: Supplementary file 8 [file Image_4.TIFF]
